# Association between Fatty Liver Index and Periodontitis: the Korea National Health and Nutrition Examination Survey

**DOI:** 10.1038/s41598-020-60797-7

**Published:** 2020-03-02

**Authors:** Ji-Youn Kim, Gyu-Na Lee, Hyun Chul Song, Yong-Moon Park, Yu-Bae Ahn, Kyungdo Han, Seung-Hyun Ko

**Affiliations:** 1 Division of Oral & Maxillofacial Surgery, Department of Dentistry, St. Vincent’s Hospital, College of Medicine, The Catholic University of Korea, 222 Banpo-daero, Seocho-gu, Seoul 06591 Republic of Korea; 2 Statistics and Actuarial Science, Soongsil University, 369 Sangdo-ro, Dongjak-gu, Seoul 06978 Republic of Korea; 3 Epidemiology Branch, National Institute of Environmental Health Sciences, National Institutes of Health, Research Triangle Park, 111T. W. Alexander Dr., Research Triangle Park, Durham, NC 27709 USA; 4 Division of Endocrinology and Metabolism, Department of Internal Medicine, St. Vincent’s Hospital, College of Medicine, The Catholic University of Korea, 222 Banpo-daero, Seocho-gu, Seoul 06591 Republic of Korea

**Keywords:** Dental epidemiology, Risk factors

## Abstract

It has been suggested that periodontitis is associated with metabolic abnormalities including non-alcoholic fatty liver disease (NAFLD). The fatty liver index (FLI) is a non-invasive surrogate marker and predictor of NAFLD. We aimed to determine whether FLI itself would be associated with periodontitis through a secondary analysis of previously reported nationally representative probability sample data of the Korean population. FLI was calculated from a previously developed algorithm which combines measures of body mass index (BMI), waist circumference, triglyceride, and gamma-glutamyl transferase (GGT). Periodontitis was diagnosed based on the Community Periodontal Index (CPI) developed by the World Health Organization. Of 4,272 participants, 26.1% were diagnosed with periodontitis. Higher FLI was associated with a higher prevalence of periodontitis (Odds ratio (OR) _highest vs. lowest quartile of FLI_,1.63; 95% confidence interval (CI), 1.23–2.16; P = 0.001 for trend) adjusting for confounding factors. In the highest FLI quartile, prevalence of periodontitis was higher in individuals with diabetes (OR _highest vs. lowest quartile of FLI_, 2.89; 95% CI, 1.01–8.27 for diabetic subgroup; OR _highest vs. lowest quartile of FLI_, 1.45; 95% CI, 1.07–1.96 for non-diabetic subgroup). In summary, FLI was associated with prevalent periodontitis.

## Introduction

Periodontitis is a dental bacteria-induced chronic inflammatory condition characterized by immunologic destruction of supporting structures of teeth^1^. Approximately half of the adult U.S. population suffers from periodontitis with data from the 2009 and 2010 National Health and Nutrition Examination Survey^2^. Associations between periodontitis and other health problems including obesity, coronary heart disease, stroke, metabolic syndrome, diabetes mellitus, and non-alcoholic fatty liver disease (NAFLD) have been already discovered^3–8^.

Fatty liver index (FLI) is an algorithm that combines measures of body mass index (BMI), waist circumference, triglyceride, and gamma-glutamyl transferase (GGT)^9^. FLI is a non-invasive surrogate marker and predictor of NAFLD^8,9^. For the definitive diagnosis of NAFLD, the ultrasonic evaluation or liver biopsy is required, while FLI may allow us to identify NAFLD in large-scale epidemiological research.

We hypothesize that FLI may be associated with periodontitis prevalence. Therefore, the objective of this study was to assess the relationship between FLI and periodontitis in a large probability sample of the Korean population using data from the 2010 Korea National Health and Nutrition Examination Survey (KNHANES). Since NAFLD accounts for 20–30% and 5–18% of the general population in Western and Asian countries^10^, respectively, it is assumable that a population group with a high prevalence of periodontitis may exist among those not diagnosed with NAFLD but possess high FLI. This hypothesis may be observable in a large population dataset such as KNHANES. Benefitting from the size of the dataset, we stratified the Korean general population according to FLI quartiles to see if the association with periodontitis was stronger in subjects with higher FLI.

## Methods

### Survey and subjects

Data of this study were obtained by secondary analysis of a previously reported nation-wide probability sample data, which was previously reported as the 2010 Korea National Health and Nutrition Examination Survey (KNHANES) study^11^. This survey was conducted by the Division of Chronic Disease Surveillance, Korea Centers for Disease Control and Prevention under Korean Ministry of Health and Welfare, Sejong, Korea^12^. Trained interviewers collected nationally representative samples based on standard household surveys^12^. From sampled household units based on population and housing census from the 2005 National Census Registry in Korea, participants were selected by using a stratified, multistage, and probability-based sampling design with proportional allocation^13^. A total of 8,473 participants were candidates in the 2010 KNHANES survey. For this study, 6352 participants aged over 19 years were included. After excluding those with cancer, liver diseases, low kidney function, pregnancy, heavy drinking, or missing data, 4,272 participants were analyzed. Informed consent was obtained from all interviewees in this survey. This study was approved by the Institutional Review Board (IRB) of the Korean Center for Disease Control and Prevention (IRB number: 2010-02CON-21-C) and was conducted according to Declaration of Helsinki guidelines. Informed consent was obtained from all participants at the time of survey collection.

### Sociodemographic and lifestyle variables

A self-administered questionnaire was used to assess sociodemographic and lifestyle variables (including smoking, alcohol consumption, physical exercise, household income, and education level) of participants. Regarding smoking status, participants were classified as current smokers or nonsmokers. Those who smoked currently and had smoked more than 100 cigarettes in their lifetime were defined as current smokers^14^. International physical activity questionnaire was used to measure the degree of physical exercise. Regular exercisers were defined as those who exercised for ≥five occasions per week for 30 minutes per session or those who participated in strenuous physical activity for ≥three occasions per week for 20 minutes per session^14,15^. Education level was categorized as above (high school education or above) or less (middle school education or less) than ten years of education. Household income was divided by the number of included family members and categorized into quartiles. The lowest quartile of household income was <USD 1,092.40 per month.

### Anthropometric and biochemical measurements

Anthropometric measurements of study participants were taken by trained staff members. Bodyweight and height were measured for subjects with indoor clothing without shoes. For waist circumference, the narrowest point between the iliac crest and the lower border of the rib cage was used. Body mass index (BMI) was calculated using the formula of weight/height^2^ (kg/m^2^). For systolic and diastolic blood pressure measurements, a standard mercury sphygmomanometer (Braumanometer; W.A. Baum Co., Inc., Copiague, NY, USA) was used. The measurement was done twice at a 5-minute interval. Venous blood was collected from the antecubital vein of each participant after a fasting period of 8 hours. Concentrations of fasting plasma glucose, total cholesterol, triglycerides, high-density lipoprotein (HDL) cholesterol, low-density lipoprotein (LDL) cholesterol, and GGT were measured from blood samples using an automated chemistry analyzer (Hitachi Automatic Analyzer 7600, Hitachi, Tokyo, Japan) and commercially available kits (Daiichi, Tokyo, Japan).

Diabetes was defined as fasting glucose higher than 126 mg/dL, current use of anti-diabetic medication, or previous physician diagnosis of diabetes. Metabolic syndrome was defined if three or more of the following criteria were fulfilled^16^: waist circumference of ≥90 cm in men and ≥85 cm in women; fasting triglyceride ≥150 mg/dL or the use of lipid-lowering medication; HDL cholesterol <40 mg/dL in men and <50 mg/dL in women or the use of associated medication; blood pressure of 130/85 mmHg or higher or current use of associated medication. Diabetes mellitus, one of obesity-related metabolic disorders that affects nearly 10% of world population^17^, is characterized by hyperglycemia caused by defects in insulin secretion, insulin action, or both^18^.

FLI is a noninvasive method of assessing hepatic steatosis predicted with body mass index (BMI), waist circumference, triglyceride, and gamma-glutamyl transferase (GGT), calculated with the following formula^9^:

$$\begin{array}{c}{\rm{FLI}}=({{\rm{e}}}^{0.953\times {\rm{loge}}({\rm{triglycerides}})+0.139\times {\rm{BMI}}+0.718\times {\rm{loge}}({\rm{GGT}})+0.053\times {\rm{waistcircumference}}-15.745})/\\ (1+{{\rm{e}}}^{0.953\times {\rm{loge}}({\rm{triglycerides}})+0.139\times {\rm{BMI}}+0.718\times {\rm{loge}}({\rm{GGT}})+0.053\times {\rm{waistcircumference}}-15.745})\times 100\end{array}$$

BMI is a determinant of obesity, with BMI > 30 kg/m^2^ being considered to be obese^19^. BMI is an indicator of body fat accumulation. However, it is not indicative of fat distribution. Therefore, sometimes waist circumference is used in combination with BMI when defining obesity^3^. Triglycerides are main constituents of body fat. They are proportionally related to obesity^20^. GGT is an enzyme found in many tissues, especially abundant in the liver^21^. Blood GGT concentration is a sensitive biomarker of obesity. It is known to be increased in obesity-related health problems such as metabolic syndrome, diabetes, hypertension, dyslipidemia, and cardiovascular diseases^22^.

Subjects were divided into quartiles according to the value of FLI, each of male and female. The cut-off values in 1st quartile are 12.4 in male (n = 376) and 4.6 in female (n = 691). The cut-off values in 2nd quartile are 26.6 in male (n = 377) and 10.7 in female (n = 692). The cut-off values in 3rd quartile are 49.6 in male (n = 377) and 26.7 in female (n = 692). And the range of values in 4th quartile are above 49.6 to 99.5 in male (n = 376) and above 26.7 to 95.6 in female (n = 691).

### Oral health behaviors and periodontal status

Daily toothbrushing frequency was included in the questionnaire. The survey also included history of a periodic dental checkup categorized into yes (at least once during the past year) or no. Community periodontal index (CPI) developed by the World Health Organization was utilized to evaluate the presence of periodontal disease^23^. Index teeth were 11, 16, 17, 26, 27, 31, 36, 37, 46, 47 according to the Federation Dentaire Internationale system. Periodontitis was defined as CPIs of 3 and 4. CPI 3 was given to cases with a shallow pocket (depth of 3.5–5.5 mm) from at least one site in one of the index teeth while CPI 4 was considered for cases with a deep pocket (depth ≥ 5.5 mm).

### Statistical analysis

Results are presented as means ± standard error or percentages (standard error). For the variables which did not follow the normal distribution, logarithmic transformation was performed, and the results are presented as geometric mean ± 95% confidence intervals (CI). The relationship between values was obtained by independent t-test for continuous variables or chi-square test for categorical variables using SURVEYMEANS or SURVEYREQ procedures. Multiple logistic regression analysis was used to evaluate the association of periodontitis with FLI using SURVEYLOGISTIC procedure. In the sub-analysis on diabetes mellitus, the quartile of FLI was same as the full model. Odds ratios (OR) and 95% CI were calculated after adjusting for potential confounders. Cofounders included in Model 1 were age and sex. Model 2 was adjusted for variables in Model 1 plus smoking, exercise, drinking, income, and education. Model 3 was adjusted for variables in Model 2 plus frequency of tooth brushing and history of a periodic dental checkup. Log transformation was applied when appropriate. Statistical analysis was performed using SAS version 9.4 (SAS Institute, Inc., Cary, NC) to account for the complex sampling design^24^. Two-sided P values of <0.05 was considered a statistically significant difference.

## Results

### Baseline characteristics of subjects with periodontitis

Of 4,272 participants, 1,113 were diagnosed with periodontitis. Table 1 details baseline characteristics of participants included in the study population by the presence of periodontitis. Periodontitis subjects were older than control subjects (53.1 vs. 41.2 years, *P* < 0.0001). They also had higher percentage of males (51.7% vs. 38.9%, *P* < 0.0001). Regarding anthropometric factors associated with obesity, the periodontitis group showed higher BMI (24.2 vs. 23.3, *P* < 0.0001), larger waist circumference (83.4 cm vs. 78.9 cm, *P* < 0.0001), higher systolic blood pressure (124.9 mmHg vs. 115.9 mmHg, *P* < 0.0001), and higher diastolic blood pressure (79 mmHg vs. 75.7 mmHg, *P* < 0.0001). When obesity-related laboratory indices were considered, the periodontitis group had higher glucose level (102.6 mg/dL vs. 93.2 mg/dL, *P* < 0.0001), higher triglyceride level (120.5 mg/dL vs. 95.7 mg/dL, *P* < 0.0001), higher total cholesterol level (192.6 mg/dL vs. 183.5 mg/dL, *P* < 0.0001), higher LDL level (118.3 mg/dL vs. 111.6 mg/dL, *P* < 0.0001), but lower HDL level (45.8 mg/dL vs. 48.9 mg/dL, *P* < 0.0001). Serum glutamic oxaloacetic transaminase level (22.3 U/L vs. 20.7 U/L, *P* < 0.0001) and glutamic pyruvic transaminase level (22.4 U/L vs. 20.4 U/L, *P* = 0.0007) were higher in the periodontitis group than those in the control group. Percentage of subjects having metabolic syndrome (42.1% vs. 21.6%, *P* < 0.0001) or diabetes (15.6% vs. 5.5%, *P* < 0.0001) was also higher in the periodontitis group than that in the control group. Additionally, individuals with periodontitis were more likely to be a current smoker (25.6% vs. 19.2%, *P* = 0.002), of the lowest quartile income (22.7% vs. 14.2%, *P* < 0.0001), and less educated (52.8% vs. 78.2%, *P* < 0.0001). However, alcohol consumption (*P* = 0.2539) and regular physical exercise (*P* = 0.9319) were similar between the periodontitis group and the control group. The periodontitis group had less frequent toothbrushing (*P* = 0.001) and less percentage of periodontal checkup during the past year (18.2% vs. 23.7%, *P* = 0.001). FLI was significantly higher in individuals with periodontitis (21.6 vs. 12.2, *P* < 0.0001).

**Table 1:** Baseline characteristics of study participants.

	Periodontitis		P
	No	Yes	
	n = 3159	n = 1113	
Age (years)	41.2 ± 0.5	53.1 ± 0.6	<0.0001
Sex (male)	38.9 (1.1)	51.7 (1.6)	<0.0001
Body mass index (kg/m^2^)	23.3 ± 0.1	24.2 ± 0.1	<0.0001
Waist circumference (cm)	78.9 ± 0.3	83.4 ± 0.4	<0.0001
Systolic blood pressure (mmHg)	115.9 ± 0.4	124.9 ± 0.9	<0.0001
Diastolic blood pressure (mmHg)	75.5 ± 0.3	79 ± 0.5	<0.0001
Metabolic syndrome (%)	21.6 (0.9)	42.1 (2)	<0.0001
Diabetes mellitus (%)	5.5 (0.4)	15.6 (1.6)	<0.0001
Hypertension (%)	21.1 (0.9)	41.9 (2.3)	<0.0001
Current smoking (%)	19.2 (0.9)	25.6 (1.5)	0.0002
Alcohol consumption (%)^a^	53 (1.3)	50.6 (1.8)	0.2539
Regular exercise (%)^b^	22.7 (1.2)	22.8 (1.8)	0.9319
Low income (lowest quartile %)	14.2 (1)	22.7 (2)	<0.0001
Low education (below high school %)	21.8 (1.2)	47.2 (2.7)	<0.0001
Tooth brushing frequency (%)			<0.0001
Once a day	8.2 (0.7)	16.9 (1.9)	
Twice a day	46.8 (1.2)	51.4 (2.1)	
Thrice a day	45 (1.3)	31.6 (2.1)	
Periodic dental checkup (%)^c^	23.7 (1.2)	18.2 (1.6)	0.0014
Biochemical measurements			
Glucose (mg/dL)	93.2 ± 0.4	102.6 ± 1.5	<0.0001
Triglycerides (mg/dL)	95.7 (93.3–98.1)	120.5 (115.9–125.3)	<0.0001
Total cholesterol (mg/dL)	183.5 ± 0.9	192.6 ± 1.2	<0.0001
LDL cholesterol (mg/dL)	111.6 ± 0.8	118.3 ± 1.2	<0.0001
HDL cholesterol (mg/dL)	48.9 ± 0.2	45.8 ± 0.4	<0.0001
Gamma-glutamyl transferase (mg/dL)	21.5 (20.9–22.1)	26.7 (25.6–27.9)	<0.0001
Glutamic oxaloacetic transaminase (U/L)	20.7 ± 0.3	22.3 ± 0.3	<0.0001
Glutamic pyruvic transaminase (U/L)	20.4 ± 0.4	22.4 ± 0.5	0.0007
Fatty Liver Index	12.2 (11.5–12.9)	21.6 (20.1–23.2)	<0.0001

Abbreviations: LDL, low-density lipoprotein; HDL, high-density lipoprotein.

^a^More than once a month during the past year, excluding heavy (>30 g/day) drinkers.

^b^Those who exercised for ≥5 occasions per week for 30 minutes per session, or those who participated in strenuous physical activity for ≥3 occasions per week for 20 minutes per session.

^c^At least once during the past year.

### FLI-related characteristics of subjects with periodontitis

Table 2 compares prevalence and control of periodontitis according to variables used to calculate FLI. The periodontitis group had more BMI-high population (≥30 kg/m^2^) than the control group (5.2% vs. 3.5%, *P* < 0.0001) but less BMI-low (<18.5 kg/m^2^) population (2.8% vs. 5.7%, *P* < 0.0001). Percentage of population with waist circumstances above the cut-off for metabolic syndrome was higher in subjects with periodontitis than that in control subjects (40.4% vs. 27.6%, *P* < 0.0001). Similarly, percentage of those with triglyceride levels above cut-off for metabolic syndrome was higher in the periodontitis group than that in the control group (37.2% vs. 23.8%, *P* < 0.0001). The periodontitis group had higher percentage of those with GGT-high (highest quartile) (35% vs. 23.3%, *P* < 0.0001) than the control group, although it had lower percentage of those with GGT-low (lowest quartile) (12.6% vs. 25.2%, *P* < 0.0001) than the control group. Baseline characteristics of the study population according to FLI quartiles are additionally described in detail in Supplementary Table 1.

**Table 2:** Fatty Liver Index variables of study participants.

	Periodontitis		P
	No	Yes	
	n = 3159	n = 1113	
**Fatty Liver Index**			<0.0001
1st quartile	31 (1)	15.9 (1.3)	
2nd quartile	25.9 (1)	22.2 (1.7)	
3rd quartile	21.8 (0.9)	27.8 (1.7)	
4th quartile	21.4 (1)	34.1 (1.9)	
**Body mass index**			<0.0001
<18.5 kg/m2	5.7 (0.5)	2.8 (0.6)	
18.5–23 kg/m2	45.4 (1.2)	34.2 (1.7)	
23–25 kg/m2	20.9 (0.8)	24.9 (1.6)	
25–30 kg/m2	24.5 (0.9)	32.9 (1.8)	
≥30 kg/m2	3.5 (0.4)	5.2 (0.9)	
Waist circumference (MS+)^a^	27.6 (1.1)	40.4 (2.4)	<0.0001
Triglycerides (MS+)^b^	23.8 (0.9)	37.2 (1.8)	<0.0001
**Gamma-glutamyl transferase**			<0.0001
1st quartile	25.2 (1.1)	12.6 (1)	
2nd quartile	27.8 (1.1)	22.1 (1.4)	
3rd quartile	23.8 (0.8)	30.2 (1.8)	
4th quartile	23.2 (1.1)	35 (1.8)	

Values are prevalence and standard error in percentage.

Abbreviation: MS, metabolic syndrome.

^a^Waist circumference of ≥90 cm in men and ≥80 cm in women.

^b^Triglyceride ≥150 mg/dL.

### Adjusted ORs of periodontitis according to FLI quartiles

Table 3 shows adjusted OR and their 95% CI from multiple logistic regression analyses for subjects with periodontitis according to FLI quartiles. After adjusting for sex and age (Model 1), adjusted OR (95% CI) was 1.731 (1.317–2.292) in the highest quartile of FLI compared to the lowest quartile. When current smoking status, regular exercise, alcohol consumption, income, and education level were further controlled (Model 2), the adjusted OR (95% CI) was 1.664 (1.267–2.185) in the highest quartile of FLI compared to the lowest quartile. With additional adjustment for toothbrushing frequency and periodontal checkup (Model 3), the adjusted OR (95% CI) was 1.632 (1.235–2.185) in the highest quartile of FLI compared to the lowest quartile.

**Table 3:** Periodontitis prevalence in multiple logistic regression models for Fatty Liver Index.

	Fatty Liver Index				
	1st quartile	2nd quartile	3rd quartile	4th quartile	P for trend
**All participants**					
Model 1	1	1.296 (0.978, 1.716)	1.481 (1.105, 1.984)	1.737 (1.317, 2.292)	<0.001
Model 2	1	1.282 (0.971, 1.694)	1.441 (1.071, 1.939)	1.664 (1.267, 2.185)	<0.001
Model 3	1	1.287 (0.972, 1.706)	1.433 (1.063, 1.932)	1.632 (1.235, 2.157)	0.001
**Non-diabetes subgroup**					
Model 1	1	1.298 (0.977, 1.724)	1.481 (1.095, 2.002)	1.545 (1.142, 2.09)	0.006
Model 2	1	1.276 (0.960, 1.694)	1.422 (1.046, 1.935)	1.470 (1.090, 1.981)	0.015
Model 3	1	1.284 (0.965, 1.708)	1.418 (1.040, 1.935)	1.448 (1.068, 1.963)	0.023
**Diabetes subgroup**					
Model 1	1	1.136 (0.329, 3.925)	1.317 (0.452, 3.837)	2.724 (0.954, 7.774)	0.007
Model 2	1	1.15 (0.347, 3.810)	1.507 (0.511, 4.444)	2.936 (1.069, 8.068)	0.003
Model 3	1	1.096 (0.325, 3.701)	1.445 (0.484, 4.315)	2.891 (1.010, 8.269)	0.004

Values are adjusted odds ratio and 95% confidence interval.

Adjustments for Model 1: age and sex;

Adjustments for Model 2: Model 1 plus smoking, regular exercise, alcohol consumption, income, and education level;

Adjustments for Model 3: Model 2 plus tooth brushing frequency and periodic dental checkup.

### Adjusted ORs of periodontitis according to FLI quartiles in the diabetes subgroup

The association of diabetes with periodontitis has been extensively studied^25^. Since higher prevalence of diabetes mellitus in the periodontitis group was demonstrated in our data (Table 1), we performed additional multiple logistic regression analyses on subgroups with or without diabetes mellitus. In the non-diabetes subgroup, adjusted ORs (95% CIs) in the highest quartile of FLI compared to the lowest quartile by Model 1, Model 2, and Model 3 were 1.545 (1.142–2.090), 1.470 (1.090–1.981), and 1.448 (1.068–1.963), respectively. In the diabetes subgroup, adjusted ORs (95% CIs) in the highest quartile of FLI compared to the lowest quartile by Model 1, Model 2, and Model 3 were 2.724 (0.954–7.774), 2.936 (1.069–8.068), and 2.891 (1.018–8.269), respectively.

### Severity of periodontitis according to FLI quartiles in the diabetes subgroup

Table 4 shows percentages of CPI 3 and CPI 4 periodontitis categorized by FLI quartiles. In the total study population, percentages of CPI 3 periodontitis in subjects with the lowest and the highest quartiles of FLI were 12.55% and 28.86%, respectively. In the case of CPI 4 periodontitis, these percentages were 0.69% and 3.31%, respectively. In the diabetes subgroup, percentages of CPI 3 periodontitis in subjects with the lowest and highest quartiles of FLI were 36.05% and 46.12%, respectively. Those of CPI 4 periodontitis were 0% and 4.48% respectively.

**Table 4:** Periodontitis prevalence according to the severity of periodontitis.

	Fatty Liver Index				
	1st quartile	2nd quartile	3rd quartile	4th quartile	P
**All participants**					
CPI 3 periodontitis	12.55 (1.32)	19.65 (1.58)	25.21 (1.97)	28.89 (1.92)	<0.0001
CPI 4 periodontitis	0.69 (0.29)	0.73 (0.21)	2.34 (0.64)	3.32 (0.97)	<0.0001
**Non-diabetes subgroup**					
CPI 3 periodontitis	11.94 (1.27)	18.81 (1.58)	23.79 (2.07)	25.40 (2.08)	<0.0001
CPI 4 periodontitis	0.71 (0.3)	0.67 (0.21)	2.46 (0.7)	3.08 (0.91)	<0.0001
**Diabetes subgroup**					
CPI 3 periodontitis	36.06 (10.82)	39.67 (8.31)	40.11 (5.72)	46.12 (4.89)	0.7254
CPI 4 periodontitis		2.23 (1.63)	1.09 (0.79)	4.48 (1.88)	

Values are prevalence and standard error in percentage.

Abbreviation: CPI, Community Periodontitis Index.

CPI 3 is given for cases with a shallow pocket (depth of 3.5–5.5 mm) from at least one site in one of the index teeth and CPI 4 is given for cases with a deep pocket (depth ≥ 5.5 mm).

## Discussion

This study used nationally representative data to determine the association of FLI with periodontitis prevalence in a large probability sample of Korean adults. As FLI increased, the prevalence of periodontitis also increased in multiple logistic regression analysis after adjusting for various previously reported confounding factors for periodontitis. Higher ORs for periodontitis were observed in the diabetes mellitus subgroup in FLI high individuals (3rd and 4th quartiles). Additionally, a more severe form of periodontitis was associated with increasing FLI and the presence of diabetes.

FLI was established as a formula to predict NAFLD, a health problem with a close relationship with obesity. NAFLD has been found in 5–30% of the general Western population^10^. Surprisingly, it has been found in 90% of obese population^26^. There is a higher prevalence of NAFLD in patients with chronic diseases such as hypertension, diabetes, and coronary artery atherosclerotic disease that are known to be obesity-related^27^. NAFLD is also associated with periodontitis^8^. In line with these previous findings, FLI showed significant association with periodontitis prevalence in our large probability sample.

Previous reports have utilized FLI to identify NAFLD, especially in extensive epidemiologic studies^28,29^. The most accurate method of hepatic steatosis measurement is magnetic resonance spectroscopy. FLI has been recently shown to have a comparable accuracy to magnetic resonance spectroscopy in predicting the presence of NAFLD^30^. It is important to note that obesity-associated outcomes are apparent in Asian population at a lower level of body fat accumulation. Indeed, it is proposed that mean FLI was lower and FLI was less accurate in NAFLD prediction in Asian studies^31^. However, in recent large-scale Asian population studies in Chinese, Japanese, and Asian-Americans in the U.S., respectively, FLI was recognized and used as a relatively predictable noninvasive diagnostic tool to study of NAFLD also in Asian populations^32–34^. Regardless of ethnicity, this is the first study to show the association of periodontitis prevalence with FLI to the best of our knowledge.

Periodontitis has been reported as one of common complications of diabetes mellitus classically^25^. Diabetes mellitus also has significant association with NAFLD^35^. Furthermore, a recent article has revealed that FLI is a predictor of diabetes mellitus in a longitudinal study^36^. In concordance, our data showed a stronger association between FLI and periodontitis prevalence in the subgroup with diabetes mellitus. The severity of periodontitis was also associated with diabetes, with higher CPI 4 periodontitis percentage in the subgroup of diabetes.

Whether the presence of periodontitis that leads to high FLI status or high FLI plays a role in the development of periodontitis remains unanswered. The interacting role of diabetes with FLI and periodontitis also remains unknown. It is an intrinsic limitation of a cross-sectional analysis such as ours. Although proinflammatory cytokine levels were unavailable to us, other researchers have already provided scientific evidences that the level of proinflammatory cytokines and reactive oxygen species are increased systemically due to periodontal pathogenic biofilms in periodontitis patients^37,38^. This inflammatory condition may lead to high FLI. On the contrary, already high FLI due to obesity-related issues may lead to the development of progression of periodontitis. This was not conclusive with our data. Interestingly, it has been recently proposed that there is a two-way relationship between diabetes mellitus and periodontitis through epidemiological and animal studies^18^. That is, diabetes is a risk factor for increased prevalence and severity of periodontitis, and vice versa.

In conclusion, the findings from this nationally representative probability sample of the Korean population support the hypothesis that FLI may be associated with periodontitis prevalence, especially in subjects with diabetes. To improve public health, active screening of periodontitis in FLI-high population may be clinically beneficial, especially in those with diabetes mellitus.

## Supplementary information

Supplementary Table 1.
