# Supplementary material for: Association between Fatty Liver Index and Periodontitis: the Korea National Health and Nutrition Examination Survey
Source: Sci Rep. 2020 Mar 2;10:3805. doi: 10.1038/s41598-020-60797-7 (PMC7051950; doi:10.1038/s41598-020-60797-7)
Supplement: Supplementary file 1 — Supplementary Table 1. [file 41598_2020_60797_MOESM1_ESM.docx]

**Association between Fatty Liver Index and Periodontitis: the Korea National Health and Nutrition Examination Survey**

Ji-Youn Kim^1^, Gyu-Na Lee^2^, Hyun Chul Song^1^, Yong-Moon Park^3^, Yu-Bae Ahn^4^, Kyungdo Han^2*^, Seung-Hyun Ko^4*^

^1^Division of Oral & Maxillofacial Surgery, Department of Dentistry, St. Vincent’s Hospital, College of Medicine, The Catholic University of Korea, 222 Banpo-daero, Seocho-gu, Seoul 06591, Republic of Korea

^2^Statistics and Actuarial Science, Soongsil University, 369 Sangdo-ro, Dongjak-gu, Seoul 06978, Republic of Korea

^3^Epidemiology Branch, National Institute of Environmental Health Sciences, National Institutes of Health, Research Triangle Park, 111 T. W. Alexander Dr., Research Triangle Park, Durham, NC 27709, USA

^4^Division of Endocrinology and Metabolism, Department of Internal Medicine, St. Vincent’s Hospital, College of Medicine, The Catholic University of Korea, 222 Banpo-daero, Seocho-gu, Seoul 06591, Republic of Korea

* Co-correspondence

Correspondence:

Kyungdo Han, PhD

Statistics and Actuarial Science, Soongsil University,

369 Sangdo-ro, Dongjak-gu, Seoul 06978, Republic of Korea

E-mail: hkd917@naver.com

Tel. 82-2-820-0440

Fax. 82-2-823-1746

Seung-Hyun Ko, MD, PhD

Department of Internal Medicine, St. Vincent’s Hospital

College of Medicine, The Catholic University of Korea

222 Banpo-daero, Seocho-gu, Seoul 06591, Republic of Korea

E-mail: kosh@catholic.ac.kr

Tel. 82-31-249-7130

Fax. 82-31-253-8898

**Supplement Table S1.** Baseline characteristics of study participants according to Fatty Liver Index quartiles

|  | Fatty Liver Index | | | |  |
| --- | --- | --- | --- | --- | --- |
|  | 1st quartile | 2nd quartile | 3rd quartile | 4th quartile |  |
|  | n=1067 | n=1069 | n=1069 | n=1067 | P |
| Periodontitis (%) | 13.2 (1.4) | 20.4 (1.6) | 27.6 (2.2) | 32.2 (2.1) | <.0001 |
| Age (years) | 35.8 ± 0.5 | 42.4 ± 0.7 | 48.8 ± 0.6 | 50.2 ± 0.7 | <.0001 |
| Sex (male) | 43.2 (2) | 39.3 (1.7) | 41.6 (1.9) | 43.3 (1.9) | 0.4379 |
| Body mass index (kg/m2) | 20.3 ± 0.1 | 22.5 ± 0.1 | 24.4 ± 0.1 | 27.3 ± 0.1 | <.0001 |
| Waist circumference (cm) | 70.3 ± 0.3 | 77.1 ± 0.2 | 83 ± 0.2 | 90.9 ± 0.3 | <.0001 |
| Systolic blood pressure (mmHg) | 109.6 ± 0.5 | 114.8 ± 0.7 | 121.9 ± 0.7 | 126.9 ± 0.7 | <.0001 |
| Diastolic blood pressure (mmHg) | 71.9 ± 0.4 | 74.5 ± 0.5 | 78.1 ± 0.5 | 81.3 ± 0.4 | <.0001 |
| Metabolic syndrome (%) | 1.7 (0.4) | 6.6 (0.8) | 30.7 (1.7) | 70.2 (1.9) | <.0001 |
| Diabetes mellitus (%) | 2.5 (0.5) | 4 (0.6) | 8.7 (1.1) | 16.8 (1.5) | <.0001 |
| Hypertension (%) | 6.5 (0.8) | 18 (1.4) | 32.9 (1.8) | 49.2 (2.2) | <.0001 |
| Current smoking (%) | 20.5 (1.8) | 18.1 (1.5) | 21.9 (2) | 22.5 (1.7) | 0.3093 |
| Alcohol consumption (%)^1^ | 53.2 (2.3) | 53.3 (2) | 51.9 (1.8) | 51.3 (1.9) | 0.8553 |
| Regular exercise (%)^2^ | 22.9 (2.2) | 22.1 (1.6) | 21.7 (1.8) | 24.1 (1.7) | 0.7786 |
| Low income (lowest quartile %) | 12.6 (1.5) | 13.5 (1.4) | 16.9 (1.7) | 22.1 (1.8) | <.0001 |
| Low education (below high school %) | 88.1 (1.4) | 77.1 (2) | 64.7 (2) | 56.9 (2.2) | <.0001 |
| Tooth brushing frequency (%) |  |  |  |  | <.0001 |
| Once a day | 8.6 (1.2) | 7.9 (1.1) | 9.9 (1.2) | 14.7 (1.8) |  |
| Twice a day | 40.6 (2.1) | 47.3 (1.8) | 52.5 (2.3) | 52.3 (1.7) |  |
| Thrice a day | 50.8 (2.1) | 44.8 (2) | 37.6 (2.2) | 33 (1.9) |  |
| Periodic dental checkup^3^ | 25.1 (2) | 22.8 (1.7) | 21.5 (1.9) | 20.1 (1.8) | 0.1668 |
| Body mass index (levels) |  |  |  |  | . |
| <18.5 kg/m2 | 17 (1.3) | 1.4 (0.4) | 0.1 (0.1) | . |  |
| 18.5-23 kg/m2 | 75.8 (1.6) | 60.5 (1.8) | 24.8 (1.4) | 4.5 (0.8) |  |
| 23-25 kg/m2 | 6.1 (1) | 30 (1.7) | 36.8 (1.9) | 16.9 (1.4) |  |
| 25-30 kg/m2 | 1.2 (0.6) | 8.1 (1.2) | 38.1 (1.9) | 62.9 (1.9) |  |
| ≥30 kg/m2 | . | . | 0.3 (0.2) | 15.6 (1.6) |  |
| Biochemical measurements |  |  |  |  |  |
| Glucose (mg/dL) | 88.7 ± 0.6 | 92.2 ± 0.6 | 97.8 ± 1.1 | 103.8 ± 1 | <.0001 |
| Triglycerides (mg/dL) | 61.6 (59.8-63.4) | 87.1 (84.5-89.7) | 116 (112.4-119.8) | 179.6 (172.3-187.2) | <.0001 |
| Total cholesterol (mg/dL) | 168 ± 1 | 180.3 ± 1.3 | 193.8 ± 1.3 | 203 ± 1.5 | <.0001 |
| LDL cholesterol (mg/dL) | 101.7 ± 0.9 | 112.1 ± 1.1 | 121.9 ± 1.3 | 118.9 ± 1.5 | <.0001 |
| HDL cholesterol (mg/dL) | 53 ± 0.4 | 49.4 ± 0.4 | 46.5 ± 0.4 | 43 ± 0.3 | <.0001 |
| Gamma-glutamyl transferase (mg/dL) | 16 (15.6-16.4) | 19.2 (18.6-19.9) | 23.5 (22.6-24.4) | 37.9 (36.1-39.8) | <.0001 |
| Glutamic oxaloacetic transaminase (U/L) | 18.9 ± 0.5 | 19.7 ± 0.3 | 21 ± 0.2 | 24.9 ± 0.4 | <.0001 |
| Glutamic pyruvic transaminase (U/L) | 14.5 ± 0.3 | 18.2 ± 0.7 | 21.4 ± 0.5 | 30.3 ± 0.8 | <.0001 |

Abbreviations: LDL, low-density lipoprotein; HDL, high-density lipoprotein.

^1^ More than once a month during the past year, excluding heavy (> 30 g/day) drinkers.

^2^ Those who exercised for ≥ 5 occasions per week for 30 minutes per session, or those who participated in strenuous physical activity for ≥ 3 occasions per week for 20 minutes per session.

^3^ At least once during the past year.
